# Separating weak integrated information theory into inspired and aspirational approaches

**DOI:** 10.1093/nc/niad012

**Published:** 2023-05-17

**Authors:** Angus Leung, Naotsugu Tsuchiya

**Affiliations:** School of Psychological Sciences and Turner Institute for Brain and Mental Health, Monash University, Wellington Road, Clayton, VIC 3800, Australia; School of Psychological Sciences and Turner Institute for Brain and Mental Health, Monash University, Wellington Road, Clayton, VIC 3800, Australia; Center for Information and Neural Networks (CiNet), National Institute of Information and Communications Technology (NICT), 1-4 Yamadaoka, Suita-shi, Osaka 565-0871, Japan; Advanced Telecommunications Research Computational Neuroscience Laboratories, 2-2-2 Hikaridai, Seika-cho, Soraku-gun, Kyoto 619-0288, Japan

**Keywords:** consciousness, integrated information theory, weak-IIT, aspirational-IIT, IIT-inspired

## Abstract

Mediano et al. (The strength of weak integrated information theory. *Trends Cogn Sci* 2022;**26**: 646–55.) separate out strong and weak flavours of the integrated information theory (IIT) of consciousness. They describe ‘strong IIT’ as attempting to derive a universal formula for consciousness and ‘weak IIT’ as searching for empirically measurable correlates of aspects of consciousness. We put forward that their overall notion of ‘weak IIT’ may be too weak. Rather, it should be separated out to distinguish ‘aspirational-IIT’, which aims to empirically test IIT by making trade-offs to its proposed measures, and ‘IIT-inspired’ approaches, which adopt high-level ideas of IIT while dropping the mathematical framework it reaches through its introspective, first-principles approach to consciousness.

Integrated information theory (IIT; Tononi 2004, Albantakis et al. 2022) distinguishes itself from other theories of consciousness by taking a first-person and first-principles approach towards identifying the physical substrate of consciousness. In this approach, IIT introspects conscious experience to identify its key aspects and then deduces the necessary physical mechanisms required to support these aspects. As a result, IIT derives a measure, a mathematical quantity (and corresponding structure) that is proposed to correspond to the quantity and quality of consciousness in a given system. Given its distinct approach, IIT makes the bold claim of being able to identify the physical substrate of consciousness using its derived measure.

IIT’s bold claim, however, is difficult to apply and test in practice. Consequently, research has focussed not on testing IIT directly, but on evaluating candidate consciousness measures inspired by the theory. In the light of this, alongside criticisms of the theory itself, Mediano et al. (2022) put forward a useful practical guide towards appealing research programmes based on the theory. They propose to separate IIT into two distinct flavours: ‘strong IIT’, which aims towards some universal formulation that can be identified with consciousness, and ‘weak IIT’, which focuses instead on developing practical measures for particular aspects of consciousness. We think this separation will be useful in guiding appealing research programmes utilizing the overall ideas of IIT. However, we also think that their overall notion of ‘weak IIT’ is too weak and should be separated out into ‘aspirational-IIT’ and ‘IIT-inspired’ approaches.

The problem we see is that much of their ‘weak IIT’ discards the framework which IIT reaches from its first-person approach. In taking a first-person approach, IIT first identifies axioms of experience, then postulates their supporting physical interactions, and finally derives a mathematical framework for measuring consciousness. Dropping this framework amounts to dropping not only the proposed framework itself but also the principled reasoning linking it and IIT’s identified axioms. As a consequence, ‘weak IIT’ may end up with measures of consciousness, which, while inspired by IIT, ultimately have nothing to do with what IIT tries to establish—a principled link between phenomenology and the physical world.

Specifically, ‘weak IIT’, as described by the authors, focuses on the ideas of information and integration, as inspired by ‘strong IIT’. However, focussing on these two ideas, in an isolated manner separate from the other core ideas proposed by IIT, is essentially arbitrary in that it lacks any theoretical reasoning beyond historical empirical evidence linking these ideas to consciousness. Why should ‘weak IIT’ ignore the other axioms of consciousness put forward by IIT, such as intrinsicality and composition (which, while not explicitly listed as axioms in IIT 2.0 or prior, were described as important aspects of the theory (Tononi 2004))? ‘Information’ and ‘integration’ can take on whole new meanings, when they are considered in isolation from the rest of the axioms. For example, one can be completely agnostic about two key axioms of IIT, ‘intrinsicality’ and ‘compositionality’. The former expresses the idea that conscious experience is intrinsic to a system, thus any integrated information must be intrinsic to the system. The latter is an idea that components of experience compose to form larger components, which is the hallmark of the rich contents of any conscious experience. In isolation from these concepts, information might be interpreted in the more common, extrinsic sense of encoding a message. But, this raises questions—‘What is *reading* this information, and what is its *meaning*?’ Likewise, integration might be interpreted along the lines of the degree of correlation among parts, rather than the degree to which a system can be considered more than just its parts. Without coming from first principles, important ideas such as these end up with different meanings. Consequently, this kind of ‘weak IIT’ is no longer IIT at all, but rather a set of proposed correlates that are inspired by IIT. The perturbational complexity index (PCI) is an example of this, as it evaluates only information and integration from an extrinsic perspective. Consequently, it is unclear how it can be extended to capture the contents of consciousness. It also requires a notion of ‘perturbation’, which at first sounds similar to, but is ultimately distinct from, IIT’s ‘perturbation’. The former is direct stimulation of the brain to evoke activity, which can be performed using the coarse method of transcranial magnetic stimulation. Meanwhile, the latter is to set a system into all its possible states in order to find its causal structure. So, while PCI’s perturbation is in the spirit of IIT, in measuring causal rather than observational properties of a system, a single, coarse-grained perturbation is not enough for inferring causal structure. For this goal, more delicate and fine methods may be required, such as optogenetics (Tononi et al. 2016, Azadi et al. 2023). PCI is already described by the authors as ‘IIT-inspired’, which we think is an appropriate description of this general approach.

Meanwhile, the dichotomous framework of ‘strong IIT’ and ‘weak IIT’ may miss a huge research opportunity, which here we will call ‘aspirational-IIT’ (Figure 1). Aspirational-IIT aims to empirically test assumptions, explanations, and predictions from IIT in a reduced form while sticking as closely to the theory’s fundamental philosophy and theoretical framework as practical. With this approach, the notion of some universal integrated information measure need not be discarded. Rather, aspirational-IIT makes required trade-offs to such a measure, which allow it to be applied in the real world. For example, efforts are being made to address specific strong IIT issues such as the so-called ‘complex search’ (Hidaka et al. 2018, Toker et al. 2019, Kitazono et al. 2020) and spatiotemporal grain of analysis (Leung and Tsuchiya 2022), as well as whether causal interactions matter over dynamical properties (Albantakis and Tononi 2019). Largely speaking, this approach is followed by most of the empirical integrated information measures listed by the authors.

**Figure 1:**
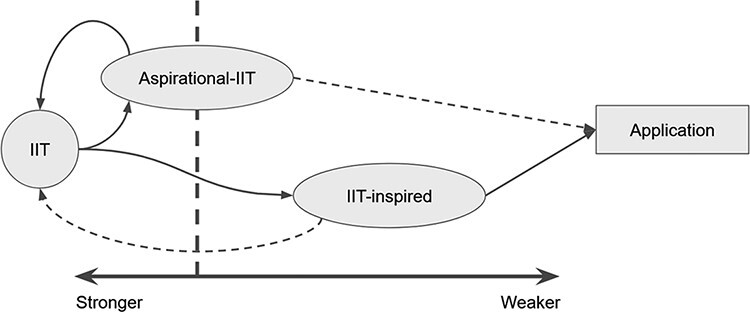
Separating ‘weak’ integrated information theory (IIT) into IIT-inspired and aspirational-IIT approaches

IIT-inspired approaches are inspired by IIT, but do not adopt the framework IIT reaches from its first-principles approach. In this manner, they are free to develop independently of the theory, with a focus on developing practical, real world measures, and discover ideas which may feed back to IIT. Aspirational-IIT approaches utilise IIT’s provided framework while leaving aside specific assumptions or requirements in order to apply it to data. Consequently, they can test specific aspects of the theory. In this sense, aspirational-IIT is stronger (i.e. closer to IIT proper) than IIT-inspired approaches. Meanwhile, they also contribute to real world application of IIT, through efforts towards addressing issues of practicality. The dashed vertical line indicates a separation of “strong IIT” and “weak IIT” put forward by Mediano et al. (2022).

From the authors’ listed empirical IIT measures, it might appear that there are no empirical measures that provide a structural (i.e. multidimensional) characterization of integrated information. Because of this, they propose an approach within their overall ‘weak IIT’, using information decomposition to characterize different ways in which information may be shared, or ‘integrated’, among system parts. The notion of information decomposition is probably useful as a general analysis tool (including in the context of comparing various empirical IIT measures). However, the notion of integration employed in this approach is different from that of IIT’s. So, while it may contribute to a new theory or framework for understanding consciousness, whether it can be linked directly back to IIT is currently unclear.

Meanwhile, aspirational-IIT would directly utilize IIT’s structural formulation of integrated information. While people generally focus on IIT’s scalar integrated information measure, it actually is a summary of IIT’s proposed ‘maximally irreducible conceptual structure’ (MICS). The MICS is hypothesized to correspond to specific conscious experiences and characterizes the information that each subpart of a system contributes (and where it contributes to). As strong IIT already provides this comprehensive structural characterization of consciousness, aspirational-IIT can adapt it for more practical use. Indeed, work along this line is already being done, with structures being estimated in neural data, and linked to both conscious contents and level, through the application of empirical integrated measures or of IIT’s own framework with clearly specified limitations (Haun et al. 2017, Leung et al. 2021). Using aspirational-IIT in this way, alongside characterizing the structure of conscious experience together with neural recordings, can allow us to test IIT’s claim linking structures of experience to structures of integrated information (Tsuchiya et al. 2016, Fink et al. 2021). In this way, aspirational-IIT allows us to create new experimental paradigms to test IIT’s framework, a benefit that IIT-inspired approaches do not supply as a consequence of them lacking direct, principled links back to IIT.

In summary, we think that ‘weak IIT’ does not need to jump so far away from strong IIT’s proposed framework. Instead, we can distinguish between IIT-inspired and aspirational-IIT. The former can develop independently from IIT, while the latter aims to more directly test IIT’s assumptions and predictions to support or refute it.

## Data Availability

No new data were generated or analysed in support of this research.
